# Mini Electrodes on Ablation Catheters: Valuable Addition or Redundant Information?—Insights from a Computational Study

**DOI:** 10.1155/2017/1686290

**Published:** 2017-05-03

**Authors:** Stefan Pollnow, Joachim Greiner, Tobias Oesterlein, Eike M. Wülfers, Axel Loewe, Olaf Dössel

**Affiliations:** ^1^Institute of Biomedical Engineering, Karlsruhe Institute of Technology (KIT), 76131 Karlsruhe, Germany; ^2^Institute for Experimental Cardiovascular Medicine, University Heart Center Freiburg, Bad Krozingen and Medical Faculty, University of Freiburg, Elsässer Str. 2Q, 79 110 Freiburg im Breisgau, Germany

## Abstract

Radiofrequency ablation has become a first-line approach for curative therapy of many cardiac arrhythmias. Various existing catheter designs provide high spatial resolution to identify the best spot for performing ablation and to assess lesion formation. However, creation of transmural and nonconducting ablation lesions requires usage of catheters with larger electrodes and improved thermal conductivity, leading to reduced spatial sensitivity. As trade-off, an ablation catheter with integrated mini electrodes was introduced. The additional diagnostic benefit of this catheter is still not clear. In order to solve this issue, we implemented a computational setup with different ablation scenarios. Our* in silico* results show that peak-to-peak amplitudes of unipolar electrograms from mini electrodes are more suitable to differentiate ablated and nonablated tissue compared to electrograms from the distal ablation electrode. However, in orthogonal mapping position, no significant difference was observed between distal electrode and mini electrodes electrograms in the ablation scenarios. In conclusion, catheters with mini electrodes bring about additional benefit to distinguish ablated tissue from nonablated tissue in parallel position with high spatial resolution. It is feasible to detect conduction gaps in linear lesions with this catheter by evaluating electrogram data from mini electrodes.

## 1. Introduction

Cardiac arrhythmias, for example, atrial fibrillation (AF), are among the most common diseases in the Western world, which cause heart failure, stroke, and increased mortality [1, 2]. Since the first use of catheter ablation for patients with AF by Haïssaguerre et al. in the 1990s, radiofrequency ablation (RFA) has become a well-established clinical procedure to treat this supraventricular arrhythmia [3–5]. The cornerstone of this minimally invasive treatment is the intentional creation of transmural and continuous ablation lesions by applying radiofrequency currents. Several imaging methods can be used to assess lesion quality, like late gadolinium-enhanced MRI or ultrasound [6, 7]. It is also feasible to indirectly estimate lesion formation by using more dynamic parameters from real-time contact force or impedance measurements [8–10]. Furthermore, clinicians are trying to evaluate acute lesions by analyzing attenuation of recorded intracardiac electrograms (IEGMs) [11, 12]. However, due to a high recurrence rate of AF (around 30–40%), many patients have to undergo a second procedure [13]. There are several reasons which may explain this relatively bad success rate. For example, incomplete ablation lines or postprocedural reconnection of myocardium can contribute to recurrence of arrhythmias [14]. Intraprocedural end points for terminating the ablation procedure of persistent AF are also not well standardized [15, 16]. Moreover, a reduced sensitivity for real-time monitoring of lesion formation leads to nontransmural lesions. Therefore, multielectrode catheters or high-density mapping catheters have been developed in order to improve the precise characterization of ablation lesions as well as arrhythmogenic tissue [17, 18]. However, the interpretation of these electrical signals is complicated, as unipolar electrograms (UEGMs) are highly sensitive for interference, for example, from ventricular far fields, and bipolar electrograms (BEGMs) are strongly dependent on the direction of the excitation wavefront. Furthermore, interventional cardiologists have to change regularly between the ablation catheter and the high-density mapping catheter to assess lesion quality during RFA procedure. This hinders reproducibility as well as robustness of measurements of IEGMs as well as a precise positioning of the ablation catheter. Therefore, a new ablation catheter with integrated mini electrodes (MEs) was introduced in 2014 (MiFi™, Boston Scientific, Natick, MA, USA). According to the manufacturer's specifications, this novel catheter design improves accurate electrogram (EGM) localization and high resolution mapping simultaneously during RFA. Furthermore, these MEs promise to allow a local lesion maturation feedback as well as identification of gaps between linear lesions. To evaluate the performance, several clinical and animal studies were performed to determine the added value of this new catheter.

Avitall et al. studied* in vivo* recorded EGMs from 4.5 mm irrigated and 8 mm nonirrigated ablation catheters with MEs in canine atria and ventricles. When maximal EGM attenuation was recorded with the MEs, transmural lesions were produced. Additionally, they confirmed lesion monitoring and improved diagnostic accuracy of these integrated MEs. However, further clinical studies are required to prove reproducibility of lesion assessment with this catheter [19].

Lloyd et al. investigated EGM characteristics of the MiFi catheter during typical atrial flutter in humans. Measured EGMs from the miniaturized electrodes had increased signal amplitudes in nonablated tissue in contrast to ablated tissue. A limitation of that study may be the imprecise positioning as well as the unstable contact of the ablation catheter above healthy and ablated tissue. Operators monitored the position of the catheter and the ablated area fluoroscopically or electroanatomically during the electrical recordings. However, this information was only partially considered when evaluating the IEGMs. Additionally, this study is restricted by the reproducibility of these minimally invasive measurements for more complex lesions, for example, linear lesions [20].

However, it remains unclear whether the MEs will provide further information about the ablation lesion, for example, geometry of the lesion or identifying a conduction gap, due to the above-mentioned limitations in both studies. Therefore, changes of EGMs from the MEs have to be investigated for different ablation scenarios under reproducible conditions in order to improve the diagnostic benefit of this catheter. A better understanding of the EGMs from the MEs may help to assess precise lesion development.

Computational simulations offer a controlled environment to study cardiac electrophysiology under physiological as well as pathological conditions, for example, myocardium with ablation lesions or arrhythmogenic substrate. Furthermore, it is feasible to determine the electrical characteristics of simulated signals by varying catheter orientation as well as catheter position.

In this* in silico* study, we investigated the diagnostic benefit of a catheter with mini electrodes for different ablation scenarios. For this purpose, electrical characteristics of simulated IEGMs from the distal electrode as well as the miniaturized electrodes were analyzed above nonablated and ablated tissues for different catheter positions. This study addresses the research question of whether we gain additional information about nonablated and ablated tissues using integrated mini electrodes instead of a typically nonirrigated 8F ablation catheter.

## 2. Methods

### 2.1. Computational Setup

Our* in silico* study was performed with finite element bidomain formulations using the cardiac simulation framework (acCELLerate) [21, 22]. An isotropic patch of myocardium with a size of 80 mm × 24 mm × 4.8 mm was initially created in an unstructured grid consisting of tetrahedral elements with edge lengths between 0.05 mm and 0.3 mm. Anisotropic tissue could deliver probably more accurate results; however, specific information about fibre orientation and anisotropy ratios are required to simulate in detail IEGM morphology. In order to reduce the number of model assumptions, we modeled isotropic tissue. We performed initial studies indicating that this simplification is justified when a small patch of myocardium is stimulated with a planar wave. An ablation lesion was incorporated into this patch by considering the cone-like temperature gradient. Briefly, this ablation lesion consisted of a necrotic core consisting of irreversibly injured myocardium as well as cells with reversible loss of excitability. The necrotic core is surrounded by a border zone representing heated myocardium with varying intracellular conductivities [23]. Myocardium surrounding the catheter as well as the ablation lesion was meshed in a finer resolution (edge length: 0.05–0.1 mm). The Ten Tusscher and Panfilov cell model was used to simulate membrane dynamics [24]. We slightly adapted the computational model of an acute ablation lesion as presented by Keller et al. and Greiner et al. for tetrahedral elements [23, 25]. Hereby, we adapted the temperature decrease from 50 to 40°C in the 3 mm wide border zone of the ablation lesion by using an exponential fit according to experimental and clinical studies [23, 26, 27]. Our* in silico* model did not consider inflammation because ablation lesions were investigated shortly after RFA procedure. Furthermore, we did not integrate edema in this model, which is observed during the RFA procedure and causes swelling of the myocardium [28].

The 8 mm nonirrigated ablation catheter was positioned either orthogonal or parallel to the myocardium. In orthogonal position, the catheter was deforming the surface of myocardium by a penetration depth of 1.2 mm (see Figure 2). In parallel position, there was no tissue deformation, but the catheter was fully contacting myocardium. An excitation wavefront was initiated by applying stimulus currents at the left tissue boundary. We adjusted intracellular and extracellular conductivities to reach a conduction velocity (CV) of approximately 800 mm/s in healthy myocardium (see Table 1), which is within the range of experimental measurements by Clayton et al. [29]. According to Greiner et al. CVs were changed in the border zones by varying intracellular conductivities (see Table 1) [25]. The cardiac patch was completely surrounded by blood with an extracellular conductivity of 0.7 S/m [30]. The thickness of the blood layer was 24.6 mm above and 6.6 mm below the myocardium. Hereby, the edge length of the tetrahedral elements in blood was varied between 0.3 and 1 mm to reduce computation time. No-flux Neumann conditions were implemented at the boundaries of the intracellular domain to blood as well as the outer edges of the simulation setup for both domains. Additionally, the extracellular potential *ϕ*_*e*_ was subjected to the uniqueness constraint (*Ω* represents the complete domain):

(1) $$\begin{array}{c} \int_{\Omega }\phi_{e}d\Omega =0. \end{array}$$

### 2.2. *In Silico* Model of the MiFi Ablation Catheter and Different Scenarios

**Figure 1:**
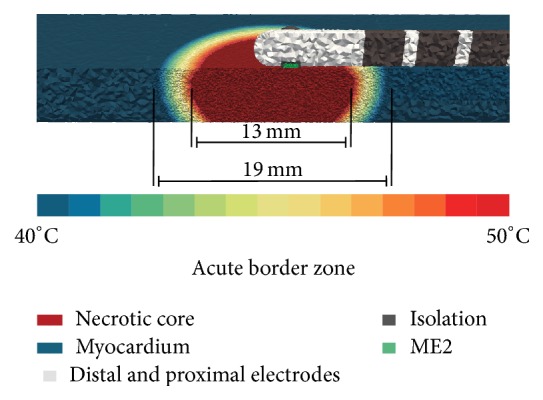
Exemplary simulation setup for parallel catheter orientation P with an 8 mm nonirrigated ablation catheter. Ablation catheter and myocardium are completely surrounded by blood. ME2 is in direct contact with the myocardium. In this simulation scenario, the ablation catheter is slightly touching the cardiac tissue. The border zone of ablation lesion is shown by a color gradient. The varying resolution of the tetrahedral grid elements with edge lengths from 0.05 to 0.3 mm can be seen.

We modeled the 8 mm nonirrigated MiFi ablation catheter according to technical specifications from the manufacturer with tetrahedrons having edge lengths between 0.05 mm and 0.3 mm. The three MEs consist of the smallest tetrahedral elements (edge lengths: approx. 0.05 mm) due to their total diameter of 1 mm (see Figure 1). MEs are completely surrounded by a thin isolation layer with no conductivity to separate them from the distal ablation electrode. Extracellular conductivity of each recording electrode was set to 7000 S/m, leading to an equipotential volume of extracellular potential [23].

**Figure 2:**
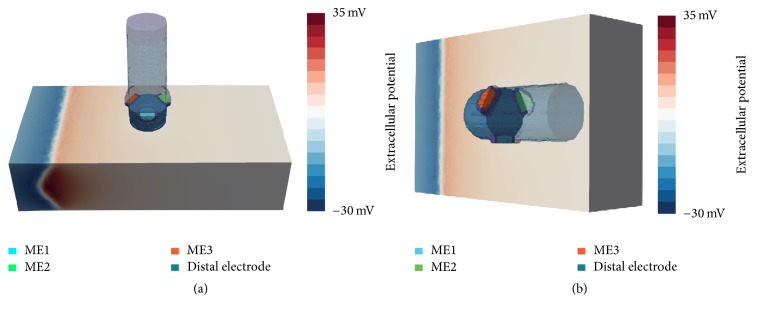
Positioning of ablation catheter and orientation of MEs for orthogonal (a) and parallel (b) orientation. (a) ME1 is oriented towards the reader, perpendicular to the propagation direction of the excitation wavefront. ME2 and ME3 are shifted by an angle of 120°, pointing away and towards the excitation wave, respectively. (b) In parallel orientation, the ablation catheter is tilted and placed flat onto the tissue patch. The myocardium and the catheter are completely surrounded by blood (not shown here). The excitation wavefront is propagating from the left tissue boundary (extracellular potentials are exemplarily shown here, depicting a moment before the excitation has passed the catheter).

To investigate signal characteristics, different mapping scenarios for this ablation catheter were implemented. For each scenario, simulated EGMs were determined for orthogonal orientation (O) of the ablation catheter. Hereby, the catheter touched the myocardium with tissue deformation (penetration depth: 1.2 mm) and all MEs were surrounded by blood due to an angle of 90° between catheter and myocardium. Considering the longitudinal axis of the catheter, ME1 was located at the side of the catheter, so that the angle between the direction of the excitation wavefront and ME1 was 90°. When turning counterclockwise by 120°, ME2 was situated at the backside of the catheter. After further 120°, ME3 was pointing towards the excitation wavefront with an angle of 30° between the longitudinal axis of the catheter and the direction of the propagation wavefront (see Figure 2).

Furthermore, each ablation scenario was performed also for parallel orientation (P) of the ablation catheter. For this purpose, the catheter was tilted by 90° along its longitudinal axis. In this position, the ablation catheter was slightly touching the myocardium (initial penetration depth: 0 mm). ME2 had full contact with the myocardium, whereas the opposite MEs were pointing towards the blood. In one scenario, we also considered various tilting angles of the ablation catheter between O and P. In the following, different mapping scenarios are briefly explained.

*Healthy Myocardium.* We placed the catheter on healthy myocardium without an ablation lesion. Figure 1 shows exemplary positioning of the ablation catheter in P. It is worth emphasizing that ME2 was directly in contact with myocardium without penetration.

*Tilting Angle.* Based on the previous scenario, we investigated signal characteristics by tilting the catheter between O and P in steps of 15° (simulation setup is shown in Supplementary Material available online at https://doi.org/10.1155/2017/1686290). The initial distance between catheter and myocardium was set to 0.6 mm in O. Therefore, a thin blood layer between catheter and myocardium without penetration was achieved with a tilting angle of 0° (P). In the following scenarios, tilting angles between catheter and myocardium were set to 90° (O) or 0° (P).

*Rotation.* Based on the first setup, we investigated simulated EGMs when rotating the catheter around its longitudinal axis. Rotation of catheter was performed in clockwise direction around its longitudinal axis for rotation angles between 0° and 360° for both orientations. In contrast to the previous scenario, ME2 had direct contact with myocardium for a rotation angle of 30°. In the following scenarios, we also used this rotation angle for determining signal characteristics of one ME directly contacting the myocardium.

*Penetration Depth.* In this scenario, we determined signal characteristics by varying penetration depth of the catheter. Hereby, penetration depth was stepwise reduced by 0.2 mm for both cases. Initial penetration depth was 0.6 mm in P and 1.2 mm in O. After the last step, the tip of the ablation catheter was slightly touching myocardium in O. However, in P, the distance between the ablation catheter and the myocardium was around 0.6 mm, resulting in a thin blood layer between the ME and the myocardium.

*Ablation Lesion with Border Zone.* The catheter was centrally positioned above an acute point-shaped ablation lesion. For achieving completely transmural lesions, width to depth ratio of ablation lesion was set to 1.25 in O and 1.6 in P. These values mimicked different ablation lesion sizes which resulted from varying contact surface of the orthogonally or perpendicularly placed catheter during the RFA procedure. The chosen ratios of the ablation lesions are within the width to depth ratios, which were reported in several* in silico* and wet lab experiments [11, 23, 31–35]. MEs were not centered to the necrotic core in P due to the distance between the tip of the ablation catheter and the MEs (around 2 mm).

**Figure 3:**
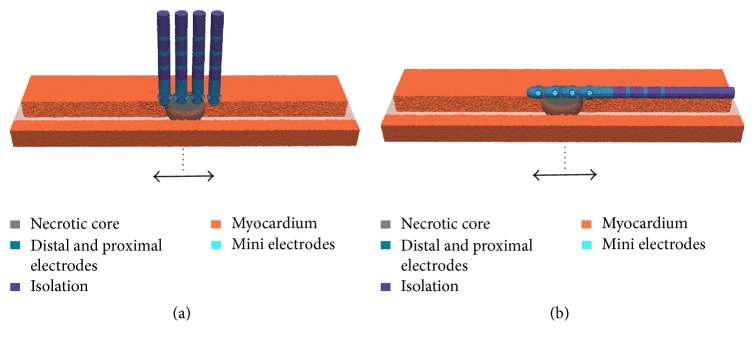
Movement of the ablation catheter over a point-shaped ablation lesion without border zone in orthogonal (a) and parallel (b) orientation (step size: 0.5–1 mm). Four different positions of the catheter are shown exemplarily. Arrows indicate the direction of movement to before and behind the lesion from the initial position (dotted line). Myocardium is more transparently shown in the direction of movement of the catheter.

*Ablation Lesion without Border Zone.* We assumed that hyperthermic characteristics of the acute lesion were cooled down after a fixed period of time. Therefore the point-shaped ablation lesion only consisted of the necrotic core without border zones (width: 13 mm). Subsequently, the ablation catheter was translated stepwise above this lesion towards the arriving excitation wavefront as well as in the opposite direction. Starting point of the movement was the central position of the catheter above the ablation lesion (see Figure 3). The maximal distance of the catheter from the central position was around 7 mm.

**Figure 4:**
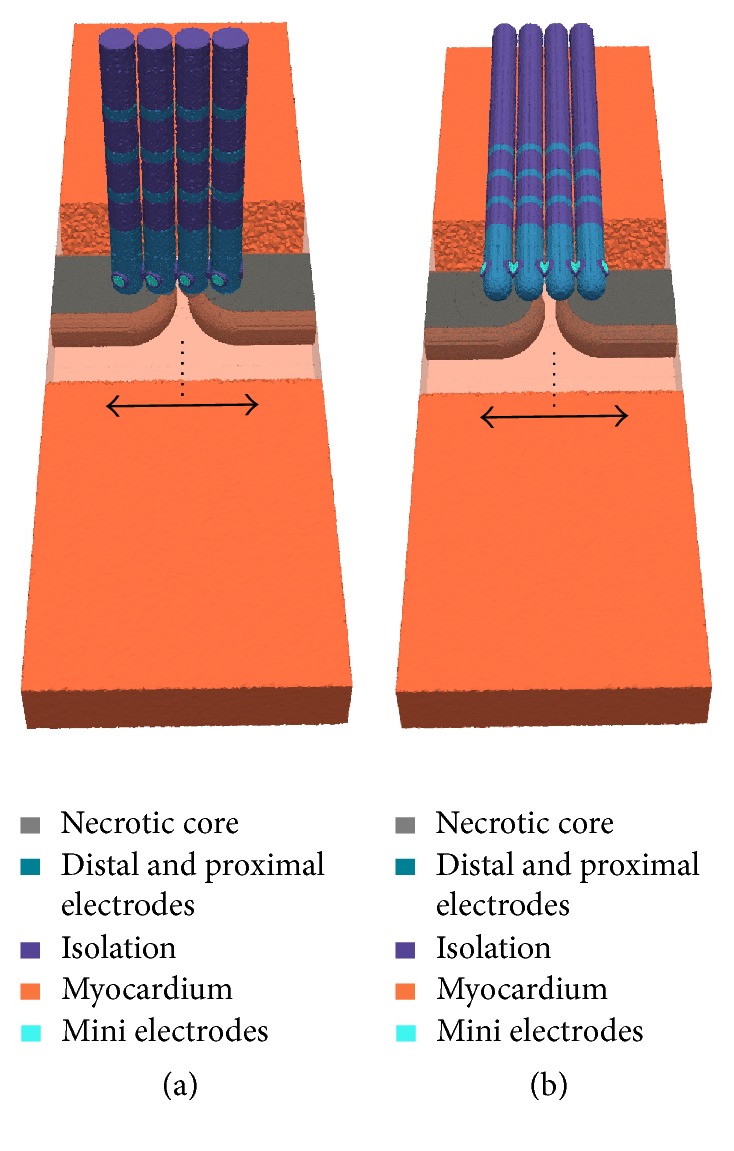
Movement of the ablation catheter along a linear lesion with a conduction gap in orthogonal (a) and parallel (b) orientation (step size: 0.5 mm). Four different positions of the catheter are shown exemplarily. Arrows indicate the direction of movement from the initial position (dotted line). Myocardium is more transparently shown in front of the linear lesion.

*Linear Lesion with Gap.* The point-shaped ablation lesion was expanded to a linear lesion having a conduction gap with a width of approximately 1.5 mm (see Figure 4). Hereby, the virtual MiFi catheter was shifted stepwise along the linear lesion (step size: 1 mm). The conduction gap represented the starting position of the catheter movement along the left side and along the right side of this linear lesion. The maximal distance of the catheter from the conduction gap was around 4 mm.

### 2.3. Calculation and Analysis of IEGMs

We used the average extracellular potential of the top 1 mm blood layer of our computational setup as reference for simulated extracellular potentials. UEGMs and BEGMs were sampled with a sampling rate of 10 kHz. For all scenarios, signal characteristics of UEGMs and BEGMs from the ablation electrodes as well as from the MEs were analyzed. BEGMs were determined by subtracting UEGMs between two adjacent MEs as well as between the distal electrode and the MEs which are counted counterclockwise starting at ME1. UEGMs were filtered according to clinical standard values using a low pass filter with a cutoff frequency of 250 Hz and a high pass filter with a cutoff frequency of 0.5 Hz. BEGMs were filtered using a 1st-order Butterworth filter with cutoff frequencies of 350 Hz (low pass filter) and 30 Hz (high pass filter). We defined local activation time (LAT) as the time point of maximum downstroke between the maximum and minimum peaks of simulated UEGMs [36].

## 3. Results

**Figure 5:**
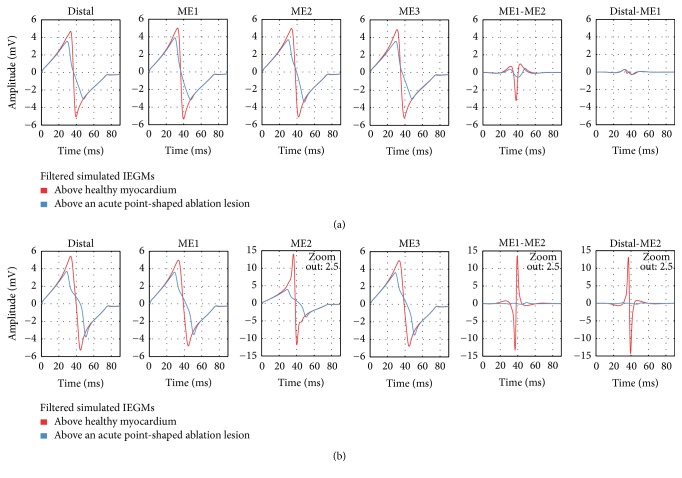
Comparison of filtered simulated IEGMs above healthy myocardium and above an acute point-shaped ablation lesion for orthogonal (a) and parallel (b) catheter orientation. Distal: UEGM of distal ablation electrode; ME1: UEGM of ME1; ME2: UEGM of ME2; ME3: UEGM of ME3; ME1-ME2: BEGM of ME1 and ME2; Distal-ME1: BEGM of distal ablation electrode and ME1; and Distal-ME2: BEGM of distal ablation electrode and ME2.

*Healthy Myocardium.* The UEGM from the distal electrode (UEGM-D) and UEGMs from the MEs (UEGMs-MEs) were determined at myocardium without ablation lesions. In O, positive peak amplitudes (around 5.0 mV) and negative peak amplitudes (around −5.0 mV) as well as the morphology of UEGM-D were in high accordance with UEGMs-MEs (see Figure 5). Only a slight increase of maximal signal amplitudes was observable for UEGMs-MEs in comparison to UEGM-D. BEGM from ME1 and ME2 (BEGM-ME1-ME2) had a peak-to-peak amplitude (*V*_pp_) of 4.2 mV. Moreover, we determined a BEGM between the distal electrode and ME1 (BEGM-D-ME1) with *V*_pp_ of 0.6 mV.

In P, UEGM from ME2 (UEGM-ME2), which was directly contacting the myocardium, had the highest signal amplitude (positive peak amplitude around 14 mV). Maximum and minimum peak amplitudes of the other UEGMs-MEs were quite similar to amplitudes of UEGM-D. *V*_pp_ of BEGM-D-ME1 was slightly increased compared to *V*_pp_ of BEGM-D-ME1 in O.

*Tilting Angle.* In this scenario, we assessed *V*_pp_ of UEGMs when reducing the tilting angle between catheter and myocardium. *V*_pp_ of each position was referenced to *V*_pp_ at a tilting angle of 90°. *V*_pp_ of UEGM-ME1 and UEGM-ME3 were increasing up to 134% compared to minimum *V*_pp_ at an angle of 90°. *V*_pp_ of UEGM-D was raised by further 13%. However, *V*_pp_ of UEGM-ME1, UEGM-ME3, and UEGM-D were smaller than 110% up to a tilting angle of 45°. At this angle, *V*_pp_ of UEGM-ME2, pointing towards the tissue, was already increased by up to 40% compared to baseline value.

**Figure 6:**
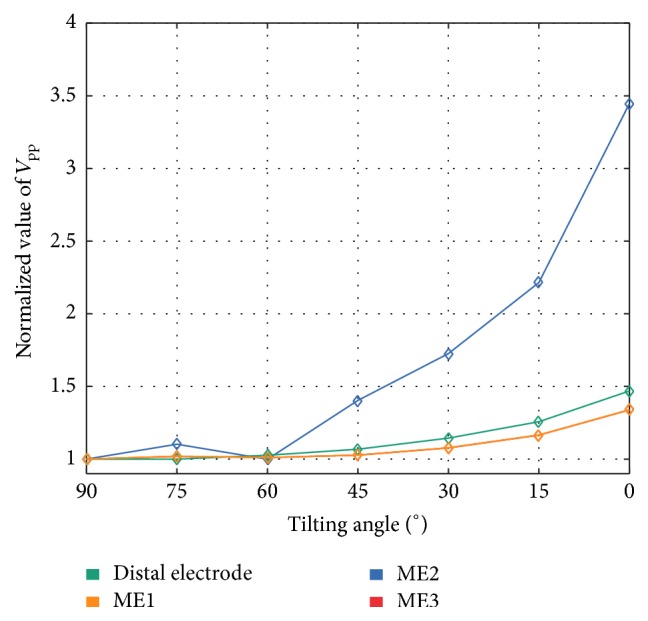
Relative changes of unipolar *V*_pp_ of the distal electrode, ME1, ME2, and ME3 by varying catheter's tilting angle with respect to myocardium. *V*_pp_ were referenced to simulated *V*_pp_ at orthogonal orientation of the catheter (90° tilting angle). Relative changes of *V*_pp_ for ME1 and ME3 were completely overlapping in all tilting angles.

In P, *V*_pp_ of UEGM-ME2, which was directly contacting the myocardium as in the previous scenario, was around 345%. Furthermore, we identified a nonlinear relation between normalized *V*_pp_ of UEGMs, especially strong in UEGM-ME2, and decreasing tilting angles (see Figure 6). Significant morphological changes in UEGM-ME1, UEGM-ME2, UEGM-ME3, and UEGM-D were not observed (not shown here).

**Figure 7:**
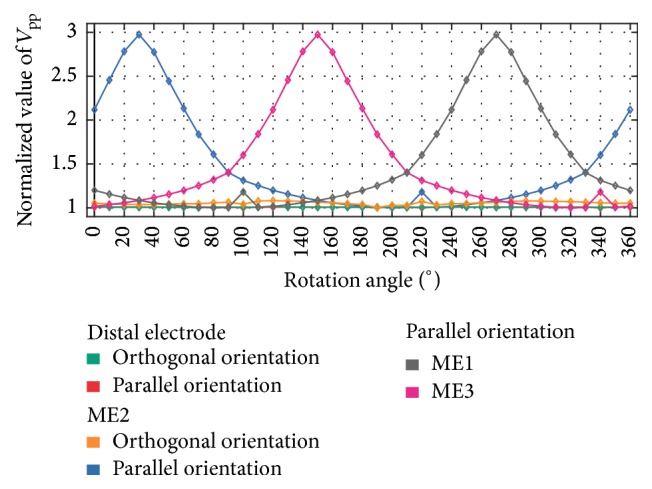
Relative changes of *V*_pp_ during rotation for distal electrode (orthogonal orientation and parallel orientation), ME2 (orthogonal orientation and parallel orientation), ME1, and ME3 in parallel orientation. The catheter is rotated above healthy myocardium. All *V*_pp_ were referenced to minimum *V*_pp_ of each electrode for complete rotation. At an angle around 30°, ME2 is in direct contact with the myocardium. Relative changes of *V*_pp_ for distal electrode are completely overlapping in both catheter positions.

*Rotation. V*_pp_ of UEGMs were assessed during rotation of the catheter above healthy myocardium. For this purpose, *V*_pp_ of UEGM-D and UEGMs-MEs were referenced to minimum *V*_pp_ of each electrode for complete rotation. Considering O, significant changes could be observed neither in UEGM-D nor in UEGMs-MEs (see Figure 7). To enhance the readability, only UEGM-ME2 was presented in this figure.

However, in P, *V*_pp_ of each ME was increasing up to a maximum *V*_pp_ when the ME directly contacted the myocardium. For example, *V*_pp_ of UEGM-ME2 was increasing up to 300% compared to minimum *V*_pp_ at an angle of 30° and further rotation led to a decrease of *V*_pp_. In contrast, *V*_pp_ of UEGM-ME3 was increasing until 150°, while its distance to the myocardium was decreased. A similar behaviour was observed for *V*_pp_ of ME2. *V*_pp_ of UEGM-D did not change significantly with increasing rotation angle.

**Figure 8:**
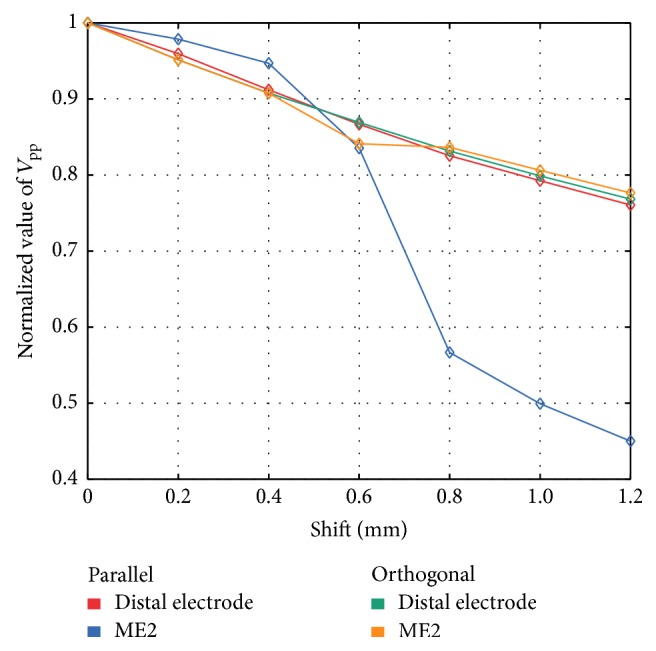
Comparison of relative changes of unipolar *V*_pp_ of the distal ablation electrode and ME2 for changing the penetration depth of the parallel placed catheter in healthy myocardium. In orthogonal orientation, relative changes of *V*_pp_ from distal electrode and ME2 were not significant.

*Penetration Depth.* We investigated the effect on *V*_pp_ for UEGM-D and UEGM-ME2 when gradually increasing the distance between the ablation catheter and the myocardium (see Figure 8). At the beginning, the perpendicularly placed catheter was penetrating the myocardium by around 0.6 mm, so that ME2 was completely surrounded by myocardium. For comparison, *V*_pp_ of each position was referenced to the initial value. *V*_pp_ of UEGM-ME2 was reduced after a shift of 0.3 mm. For the lowest penetration depth of −0.6 mm in P (distance between catheter and myocardium was 0.6 mm), *V*_pp_ of UEGM-ME2 was further reduced by around 32% compared to the change of *V*_pp_ of UEGM-D by around 23%. *V*_pp_ of UEGM-D and UEGM-ME2 were reduced by around 21% compared to baseline value when decreasing penetration depth in O.

*Ablation Lesion with Border Zone.* In this setup, UEGM-D and UEGMs-MEs were compared before and after RFA procedure in O and P. The resulting UEGMs and BEGMs are shown in Figure 5. After the RFA procedure, positive and negative peak amplitudes of UEGMs-MEs and UEGM-D were reduced by 25% and by 40%, respectively. BEGM-ME1-ME2 showed a reduction of negative peak amplitude by 85%. There was no change in BEGM-D-ME1.

In P, negative peak amplitude of UEGM-ME2 (directly in contact with myocardium) was attenuated by 70% (see Figure 5). UEGM-ME1 and UEGM from ME3 (UEGM-ME3), which were completely surrounded by blood, showed similar behaviour as UEGM-D. A strong reduction of *V*_pp_ of BEGM-ME1-ME2 was observed. In contrast to O, significant change of the BEGM from the distal electrode and ME2 (BEGM-D-ME2) occurred before and after RFA procedure. Considering signal morphology, UEGMs after RFA procedure exhibited a more flat negative slope between the signal extrema.

**Figure 9:**
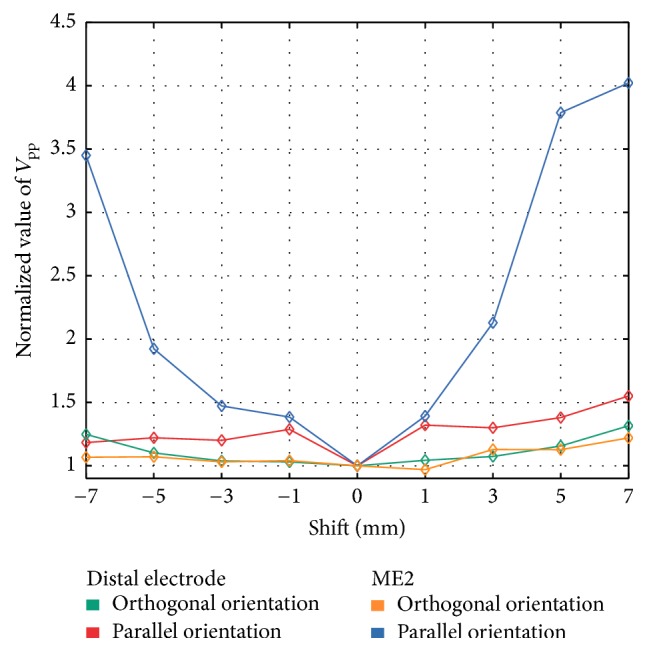
Changes of unipolar *V*_pp_ of the distal electrode (orthogonal orientation and parallel orientation) and ME2 (orthogonal orientation and parallel orientation) when the catheter was moved above the point-shaped ablation lesion. *V*_pp_ were referenced to simulated *V*_pp_ at the central position of the catheter (0 mm shift). Negative sign represents a shift towards the propagating excitation wavefront and positive sign represents a shift in the opposite direction.

**Figure 10:**
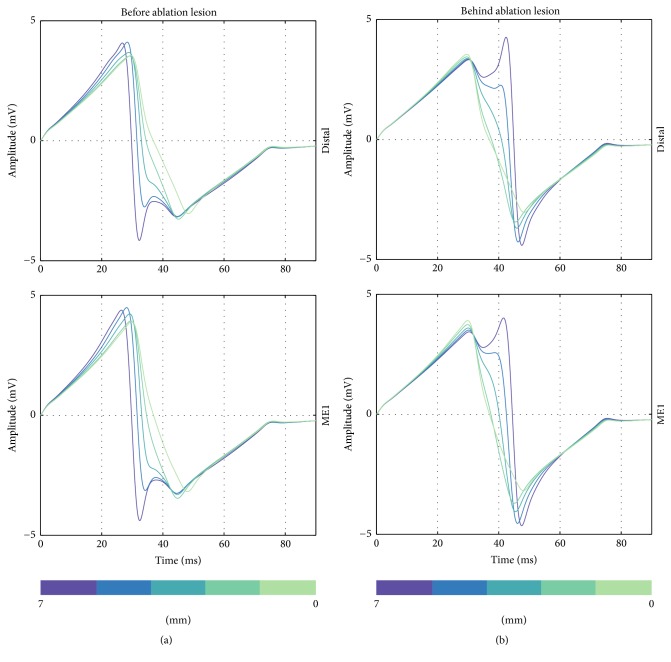
Unipolar EGMs from the distal electrode and ME1 when the catheter is orthogonally moved to the front of the point-shaped ablation lesion (a) and behind the lesion (b). Distance of catheter from center of ablation lesion is illustrated in the figure (from 7 mm to 0 mm).

**Figure 11:**
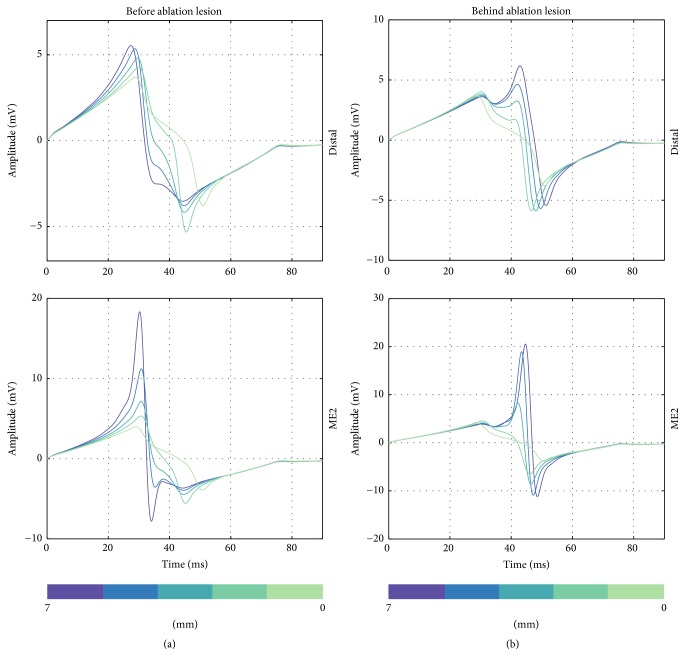
Unipolar EGMs from the distal ablation electrode and ME2 when the catheter is perpendicularly moved to the front of the point-shaped ablation lesion (a) and behind the lesion (b). Distance of catheter from center of ablation lesion is illustrated in the figure (from 7 mm to 0 mm).

*Ablation Lesion without Border Zone.* The ablation catheter was moved in steps of 1-2 mm above the point-shaped necrotic core towards the arriving wavefront (negative sign) and in opposite direction (positive sign). Relative changes of *V*_pp_ as well as signal characteristics from UEGM-D and UEGM-ME1 due to the shift of the catheter are shown in Figures 9–11. In terms of relative *V*_pp_, determined *V*_pp_ of each step changed with respect to central position above the point-shaped lesion. In O, changes of *V*_pp_ of UEGM-D and UEGM-ME1 were negligible when moving the catheter forward and backward. In a maximal distance of 7 mm, *V*_pp_ of UEGM-D and UEGM-ME1 were increased by around 22%. However, differences of *V*_pp_ between these electrodes were less than 10%.

In P, significant differences in behaviour of UEGM-D and UEGM-ME2 were observed. For UEGM-D, a relative increase of *V*_pp_ by around 28% occurred after a shift of 1 mm (*V*_pp_ raised from 7.5 mV to 9.6 mV). A further shift of the ablation catheter did not influence *V*_pp_ of UEGM-D. In contrast, *V*_pp_ of UEGM-ME2 increased by 37% (*V*_pp_ raised from 7.9 mV to 10.9 mV) in a distance of 1 mm from the central position. Compared with UEGM-D, *V*_pp_ of UEGM-ME2 was significantly increasing when the catheter was moved further.

Considering signal morphology, UEGM-D and UEGM-ME1 are shown in Figure 10. Depending on the position of the orthogonally orientated catheter, LAT and negative peak amplitudes of UEGMs were shifting by around 10 ms, when the catheter was moved before or behind the lesion. Furthermore, extra peaks were observable in positive as well as in negative peak amplitudes of UEGMs. Similar morphological changes of UEGMs could be observed in parallel orientation of the catheter (see Figure 11).

**Figure 12:**
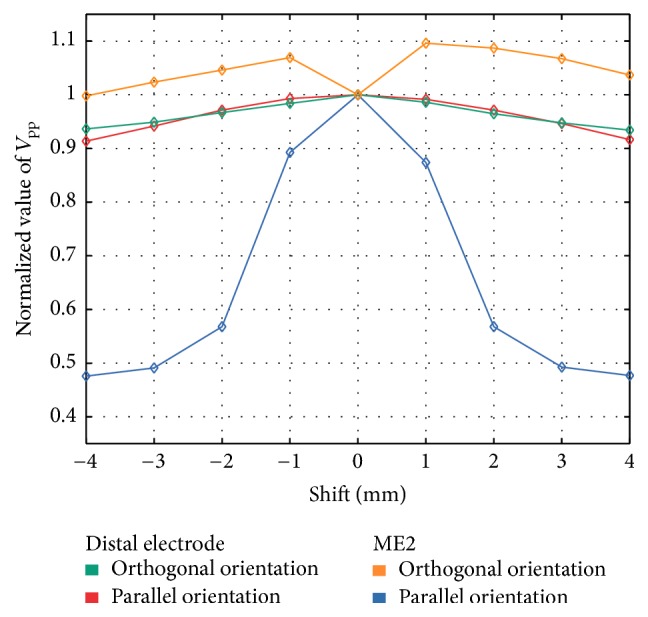
Relative changes of *V*_pp_ from the distal electrode (orthogonal orientation and parallel orientation) and ME2 (orthogonal orientation and parallel orientation) when the catheter was moved above the linear lesion. *V*_pp_ was referenced to *V*_pp_ at the conduction gap within the linear lesion (0 mm shift).

*Linear Lesion.* We assessed changes of UEGMs when the ablation catheter was moved step-by-step above a linear lesion with a conduction gap. For each step, relative changes of *V*_pp_ of UEGM-D, UEGM-ME1 in O, and UEGM-ME2 in P were referenced to the initial position of ablation catheter above the conduction gap (see Figure 12). In O, negligible changes of *V*_pp_ from UEGMs were observable when the ablation catheter entered the conduction gap (less than 2%). After a distance of 4 mm, *V*_pp_ was only reduced by around 10% compared to baseline value.

In P, relative changes of *V*_pp_ of UEGM-D could hardly be observed. However, already at a distance of 1 mm, a remarkable decrease of *V*_pp_ by around 45% (attenuation of *V*_pp_ by 8.8 mV) was detected in UEGM-ME2.

## 4. Discussion

In this* in silico* study, we investigated the diagnostic benefit of the MiFi catheter with integrated MEs for different ablation scenarios. For this purpose, we determined signal characteristics of UEGMs and BEGMs from the distal electrode as well as the MEs, when the catheter was positioned perpendicular or parallel above healthy myocardium, a point-shaped acute ablation lesion, an ablation lesion without a border zone, and a linear lesion with a conduction gap.

The developed computational setup allowed us to reproduce signal amplitudes as well as typical signal morphology (RS-morphology) of UEGMs and BEGMs, as measured in electrophysiological studies. Although the three MEs were completely surrounded by a large, highly conductive distal electrode forming a large equipotential surface (length: 8 mm; diameter around 2.7 mm), MEs in close contact with the myocardium were measuring electrical activity with high spatial resolution. The transition from parallel to orthogonal catheter orientation increases the distance between the myocardium and the MEs, which are integrated in the tip of the distal electrode. Therefore, the possible benefit of the three MEs is expected to diminish with increasing catheter angle. In general, the simulated UEGMs-MEs had higher amplitudes compared to the distal electrode, especially in parallel orientation, due to their limited field of view as well as their reduced spatial averaging effects (diameter of MEs: 1 mm).

In a scenario with healthy myocardium, we showed that the *V*_pp_ of the simulated UEGMs from integrated MEs were strongly dependent on the distance between ME and myocardium. Furthermore, tilting angles of the ablation catheter smaller than 60° were significantly influencing *V*_pp_ of the ME, which was directly facing the cardiac patch. The orientation of the catheter with a tilting angle below 60° cannot be estimated robustly with the distal electrode as well as the MEs pointing towards the blood due to the moderate changes of *V*_pp_. Furthermore, it is not conceivable to determine catheter orientation between 90° and 60° with all electrodes in a clinical scenario without an electroanatomical mapping system. In order to investigate fundamental changes in signal characteristics, we decided to consider maximal distinguishable tilting angles and therefore maximal changes in *V*_pp_ in the different scenarios. During rotation of the catheter in P, a strong increase/decrease of *V*_pp_ could be observed when a ME was pointing towards/away from the tissue patch, respectively. The voltage amplitude was also shown to be strongly dependent on the penetration depth of the ablation catheter. This sensitivity in comparing *V*_pp_ between the MEs may help to determine a defined contact and orientation of the catheter in clinical measurements.

In a scenario with an acute ablation lesion, we analyzed the relative changes of UEGMs and BEGMs to assess lesion formation during an RFA procedure. In O, a similar attenuation of *V*_pp_ of UEGM-D as well as UEGMs-MEs was determined after the creation of a transmural point-shaped ablation lesion. In clinical measurements, these differences in signal characteristics between distal electrode and MEs are negligible. Therefore, it is not feasible to gain further information about a point-shaped ablation lesion by analyzing UEGMs-MEs in this catheter position. We point out that this result is expected due to the increased distance between myocardium and MEs. Out of scope, it remains to be examined whether varying lesion transmurality leads to significant changes in UEGMs-MEs. However, in P, the reduction of *V*_pp_ was strongly significant for UEGMs-MEs, which were in direct contact with the myocardium. Simulated UEGMs-MEs were reduced by around 80% after the RFA procedure. The attenuation of these EGMs is in accordance with the studies from Avitall et al. and Price et al. [19, 37]. BEGM-ME1-ME2 was reduced by around 85% in O and nearly completely in parallel position. This criterion could be used in clinical measurements to finish the ablation procedure at a specific location assuming lesion transmurality. Moreover, in P, changes in BEGM-D-ME2 can also be used to assess lesion formation during RFA procedure. Lloyd et al. also observed a strong reduction of amplitude of bipolar EGMs from MEs above ablated and nonablated tissues (around 90%). However, in this study, catheter orientation as well as catheter position was not clearly specified, when evaluating maximal EGM attenuation from the MEs [20].

In a further scenario, we evaluated the benefit of the MEs when the ablation catheter was moved stepwise before and behind a point-shaped ablation lesion. Considering the relative changes of *V*_pp_ of UEGMs during the movement of the catheter, there was no strong difference between the distal electrode and the MEs in orthogonal position. Moreover, the ablation catheter had to be moved about 5 mm away from the central position of the lesion for measuring relative changes of *V*_pp_ around 10%. We assume that a clear separation of nonablated and ablated myocardia with this catheter orientation is not feasible in a clinical scenario. Like in the previous scenario, the potential benefit of the MEs disappeared due to their enlarged distance to the myocardium. However, in P, a significant change of *V*_pp_ by 40% occurred, when the ME was shifted from ablated tissue towards nonablated tissue due to their improved spatial resolution. A sharp increase of *V*_pp_ of UEGM-ME2 was measured, when the catheter was moved more than 5 mm in front of the center of the lesion and subsequently crossed the lesion border. A similar behaviour was observable when the ablation catheter crossed the posterior region of the point-shaped lesion after 3 mm. Hereby, the MEs directly contacting the myocardium may be used to determine the geometry of the point-shaped lesion after the RFA procedure. Moreover, when the ablation catheter was positioned orthogonally or parallel in front of the lesion, an extra peak was observable in the negative peak of the simulated UEGMs. This can be explained by the lesion influencing the propagation of the excitation wavefront and causing field distortions. A similar effect was observable in the positive peak of the UEGMs when the catheter was placed behind the lesion [23]. However, it remains highly questionable whether these morphological artifacts can be robustly detected during clinical measurements. For this purpose, a high signal-to-noise ratio as well as a stable positioning of the catheter is absolutely necessary. Hence, we conclude that it is only feasible to differentiate ablated tissue from nonablated tissue in parallel position of the MiFi catheter by analyzing *V*_pp_ of UEGMs from the ME being in direct contact with the myocardium. However, significant changes in these amplitudes will identify the transition region between ablated and nonablated myocardia. In order to achieve that, first, the lesion center has to be determined and, subsequently, the perpendicularly positioned catheter has to be shifted over the lesion region.

In a computational scenario with a linear lesion having a conducting gap of 1.5 mm, we determined the additional diagnostic benefit of this ablation catheter for detecting the gap. In orthogonal position, the relative change of *V*_pp_ was smaller than 10% for UEGM-D. Similar to the previous scenario, the MEs offered no additional spatial information about the condition of the tissue due to their increased distance to the myocardium. Therefore, robust detection of the conduction gap within the linear lesion was not feasible by analyzing *V*_pp_ of UEGM-D as well as UEGMs-MEs. For parallel position, relative *V*_pp_ changed significantly when the catheter was moving above this gap. These results support the notion that it is feasible to identify conduction gaps between linear lesions by shifting the catheter and evaluating *V*_pp_ of UEGMs from integrated MEs. However, this diagnostic benefit is only relevant when MEs are in close contact with the myocardium. MEs which are surrounded by blood are losing their improved spatial resolution. Therefore, the clinician has to consider orientation of the catheter during the RFA procedure. Our results are in agreement with the clinical studies from Price et al. also studying the ability of the catheter to detect conduction gaps [37].

The following section briefly concerns limitations of our* in silico* study. Firstly, when analyzing simulated and clinical EGMs, the filter settings of the electrical measurement system have to be regarded. It has to be pointed out that these filter settings are strongly influencing *V*_pp_ as well as signal morphology of UEGMs as well as BEGMs [23]. Therefore, in this* in silico* study, the filter settings (high pass cutoff frequency: 0.5 Hz; low pass cutoff frequency: 250 Hz) have been chosen according to common clinical values, so that they are not strongly influencing signal characteristics. For comparing these simulated EGMs with clinical EGMs recorded at a specific institution, adapting the filter settings may be required.

Secondly, differences in signal characteristics, especially in *V*_pp_, between simulated and clinical EGMs are expected due to the following reasons: (i) precise and stable positioning of catheter being not feasible in a contracting heart, (ii) reduced contact force of the ablation catheters, (iii) curved and uneven surfaces of the heart, (iv) strong heterogeneity of myocardium, (v) fibrotic tissue, (vi) neglection of mechanoelectric coupling, and (vii) ischemia due to penetration.

Moreover, spatial dimensions of our* in silico* setup have to be considered. In clinical measurements, it is not feasible to change the penetration depth of the ablation catheter or the movement of catheter with a step size from 0.1 to 1 mm. However, this study focused on the fundamental changes of signal characteristics by varying position and orientation of the catheter. It can be assumed that smaller changes of UEGMs or BEGMs cannot be robustly detected in clinical scenarios.

For validating our computational setup, the predicted data of this* in silico* model have to be compared with similar scenarios of* in vivo* experiments which have to be acquired in future studies. Subsequently, more complex ablation scenarios could be analyzed with this developed computational setup. Moreover, it is conceivable to integrate fibrotic tissue in our setup to determine further diagnostic potential of the MiFi catheter in these scenarios. These investigations could help to reduce ablation time as well as excessive destruction of myocardium and may improve lesion assessment by evaluating electrogram characteristics during the RFA procedure.

## 5. Conclusions

The main purpose of this* in silico* study was to assess whether or not an ablation catheter with additional MEs (e.g., the MiFi catheter) offers additional diagnostic benefit during an RFA procedure. In orthogonal position, it was basically feasible to distinguish between ablated and nonablated tissues by analyzing the EGMs from the distal electrode as well as the MEs. However, significant differences of EGMs between these electrodes were not identified and therefore more detailed assessment of different lesion geometries appears unlikely in clinical scenarios. However, in parallel position, the signal characteristics of unipolar EGMs from the MEs are more strongly influenced by ablated tissue and lesion geometry compared to EGMs of the distal electrode in particular when the ME is touching myocardium. Moreover, a conduction gap within a linear lesion could be identified with this catheter in parallel position.

In conclusion, the MiFi catheter yields additional diagnostic benefit to differentiate ablated tissue from nonablated tissue as well as specific lesion geometries in parallel mapping position. We could not show that clinicians will gain more information about the lesion geometry with the integrated MEs when the catheter is placed orthogonally on the cardiac tissue. With our computational setup, it is also feasible to investigate more complex ablation scenarios and arrhythmogenic tissue, for example, fibrosis. This will allow assessing further the diagnostic benefit of this catheter design.

## Supplementary Material

In this scenario, the ablation catheter was initially placed in orthogonal orientation (O) at healthy myocardium (tilting angle 90°). To achieve gentle contact between catheter and myocardium in parallel orientation (P, tilting angle 0°), the distance between myocardium and catheter was set to 0.6 mm in O. We varied the tilting angles between 90° (O) and 0° (P) in steps of 15°. Similar to the scenario “Healthy myocardium”, ME2 was directly in contact with the myocardium at a tilting angle of 0°.

## Figures and Tables

**Table 1:** Overview of intracellular and extracellular conductivities and CV for several temperature ranges. The intracellular conductivity in the border zones was exponential and is given as the range of values in this zone. We assumed a specific membrane capacity *C*_*m*_ of 0.1 *μ*F/cm^2^ and a surface to volume ratio *β* of 100 mm^−1^ for the myocardium [25].

Material	*σ*_*i*_ (S/m)	*σ*_*e*_ (S/m)	CV (mm/s)
Myocardium	0.40	0.264	800
Blood	—	0.70	—
Lesion (40–43°C)	0.47–0.53	0.264	844–873
Lesion (43–46°C)	0.53–0.36	0.264	873–784
Lesion (46–50°C)	0.36–0.01	0.264	784–160
Lesion (necrotic)	—	0.10	—
Electrode	—	7 · 10^3^	—
Isolation	—	10^−10^	—
