# Supplementary material for: Mini Electrodes on Ablation Catheters: Valuable Addition or Redundant Information?—Insights from a Computational Study
Source: Comput Math Methods Med. 2017 May 3;2017:1686290. doi: 10.1155/2017/1686290 (PMC5434470; doi:10.1155/2017/1686290)
Supplement: Supplementary file 1 — In this scenario, the ablation catheter was initially placed in orthogonal orientation (O) at healthy myocardium (tilting angle 90°). To achieve gentle contact between catheter and myocardium in parallel orientation (P, tilting angle 0°), the distance between myocardium and catheter was set to 0.6 mm in O. We varied the tilting angles between 90° (O) and 0° (P) in steps of 15°. Similar to the scenario “Healthy myocardium”, ME2 was directly in contact with the myocardium at a tilting angle of 0°. [file 1686290.f1.pdf]

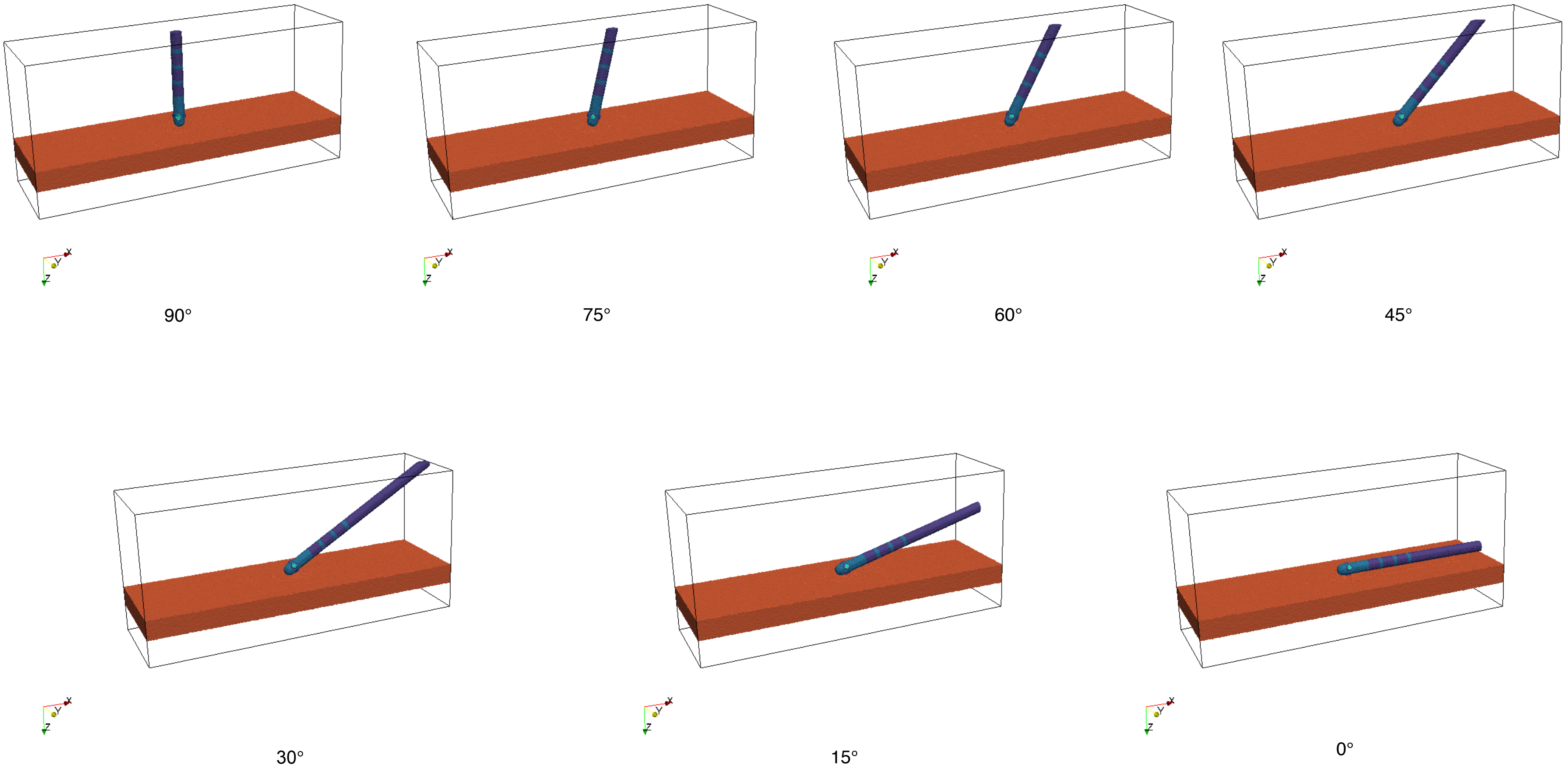


**SUPPLEMENTARY FIGURE 1:** Varying tilting angle between catheter and healthy myocardium between 90° and 0°. The excitation wavefront is propagating from the left tissue boundary (electrical activity is not shown here).
**Legend:** distal and proximal electrodes (■), isolation (■), myocardium (■), mini electrodes (■).
